# Evaluation of a Dielectric-Only Transmitarray for Generating Multi-Focusing Near-Field Spots Using a Cluster of Feeds in the Ka-Band

**DOI:** 10.3390/s21020422

**Published:** 2021-01-09

**Authors:** Álvaro F. Vaquero, Marcos Rodríguez Pino, Manuel Arrebola, Sérgio A. Matos, Jorge R. Costa, Carlos A. Fernandes

**Affiliations:** 1 Group of Signal Theory and Communications, University of Oviedo, 33203Gijón, Spain; mpino@uniovi.es (M.R.P.); arrebola@uniovi.es (M.A.); 2 Departamento de Ciências e Tecnologias da Informação, Universitário de Lisboa (ISCTE-IUL), 1649-026 Lisbon, Portugal; Sergio.Matos@iscte-iul.pt (S.A.M.); jorge.costa@iscte-iul.pt (J.R.C.); 3 Instituto de Telecomunicações, Instituto Superior Técnico, Universidade de Lisboa, 1049-001 Lisbon, Portugal; carlos.fernandes@lx.it.pt

**Keywords:** dielectric transmitarray, 3D printing, near-field focusing, near-field multi-focusing

## Abstract

A transmitarray antenna is evaluated to generate a multi-focusing spot area in the Fresnel region of the antenna in the Ka-band. The antenna is designed to focus the radiated field at a certain point using a central feeding configuration. The number of feeds is increased to create as many focusing spots as feeds. The feeds are placed along an arc defined in the principal planes of the transmitarray, radiating independent near-field spots and providing a solution with a wide-angle spot scanning without an antenna displacement and a high isolation between feeds. To validate this concept, a transmitarray based on dielectric-only cells is designed and simulated under full-wave conditions. Then, this design is manufactured using a 3D printing technique, and the prototype is measured in a planar acquisition range. Measurements are performed for different feed positions in order to validate the multi-focusing capability of the antenna. Measurements and simulations show a high agreement and validate the proposed design technique.

## 1. Introduction

Throughout the last few decades, near-field focusing (NFF) techniques have risen in popularity as an approach to concentrate the power density of the antenna on a point within the outer boundary $2D^{2}/\lambda$, where *D* is the antenna aperture. NFF antennas have received great interest in several application such as radio frequency identification (RFID) [1,2], microwave imaging [3,4], or medical hyperthermia [5,6]. Recent applications such as wireless power transfer (WPT) or 5G communications have led to emerging needs for high efficiency wireless communications between multiple devices and sensors, mostly closely distributed and within the near-field region of their antennas. Either NFF or multi-focusing techniques are preferred to improve these application performances since they should only radiate energy wherein the devices are located, increasing the efficiency.

A popular approach in NFF design is the conjugate phase (CP) used to adjust the phase of the antenna elements such that the radiated wave is in phase at a single focal point [7,8]. However, when dealing with multi-focusing solutions, other approaches may be used. A classical solution for planar arrays is to use optimization techniques to minimize a cost function with requirements for all the spots simultaneously; nonetheless, the increase of the computational cost regarding the CP and the complexity and high insertion losses of the feeding networks makes this approach unsuitable in the millimeter band.

Recently, spatially-fed antennas, such as reflectarrays or transmitarrays (TAs) [9,10], phased arrays [11], or Fresnel zone plates (FZPs) [12], have been considered to overcome these limitations, taking advantage of their capability to shape the incoming phase front of a conventional antenna. Hence, a multibeam pattern can be accomplished through selecting the feed appropriately. In [13], a graded index lens is fed by a multiple-port stacked-patch feed, achieving four different and independent beams. These solutions demand the design of a dedicated feed, not a conventional one. A straightforward approach is based on a feed displacement using a mechanically movable feed to obtain multiple independent beams, the direction of which is determined by the feed position. This solution has been successfully applied to transmitarrays and reflectarrays for space communications [14,15,16]. However, when dealing with near-field multi-focusing applications, this type of solution is not used, and published works design transmitarrays, reflectarrays, or metasurfaces using different optimization techniques, which have to include the requirement of each beam in the cost function, therefore increasing the complexity and the computational cost of the design. More recently, in [17,18], two dielectric transmitarrays were proposed to obtain multiples beams. The first one obtains a far-field multibeam solution by using a single feed and a mechanical rotation of the transmitarray itself. Then, in [18], a multi-focus transmitarray was proposed to obtain four focused spots simultaneously. The design technique was based on dividing the antenna into several areas, and each of them is associated with a different spot. This approach is suitable when a large aperture can be employed and spots are far from each other (broad angular deviation), but this is not the case.

In this work, a planar dielectric-only transmitarray is evaluated for near-field multifocusing applications in the Ka-band. The transmitarray design process is based on the analytical approach of the conjugate phase for a single-center feed configuration. Then, alternative to a mechanical displacement of the feed, the transmitarray is illuminated by a cluster of horn antennas working at 28 GHz, but different shifted carriers or switching the horn antennas in different time slots, avoiding any interference between beams. Thus, the number of focused spots obtained is equal to the number of feeds. The position of each feed must be selected properly to minimize the differences between the different incident fields, so the transmitarray behaves similar to all the feeds. In this case, the feeds are proposed to be placed on a spatial distribution according to the arc defined by the central feed and the main planes of the reflectarray. In order to validate this purpose, the design of the transmitarray is carried out using dielectric-only cells, which is evaluated in a full-wave simulation placing the horn antenna at different positions. Then, the transmitarray is manufactured using a 3D printed technique, obtaining a low-cost prototype that is measured in a planar range facility to evaluate the different focused spots. Finally, measurements and simulations show a good agreement and validate the proposed technique to easily obtain a low-cost dielectric-only transmitarray for multi-focusing near-field applications, such as near-field radars for sensing as having different spots enables examining different points on a surface without an antenna displacement. Additionally, this solution can be applied for efficient wireless power transfer and information connections between devices placed close to each other.

## 2. Near-Field Multi-Focusing Spot Generation

**Figure 1:**
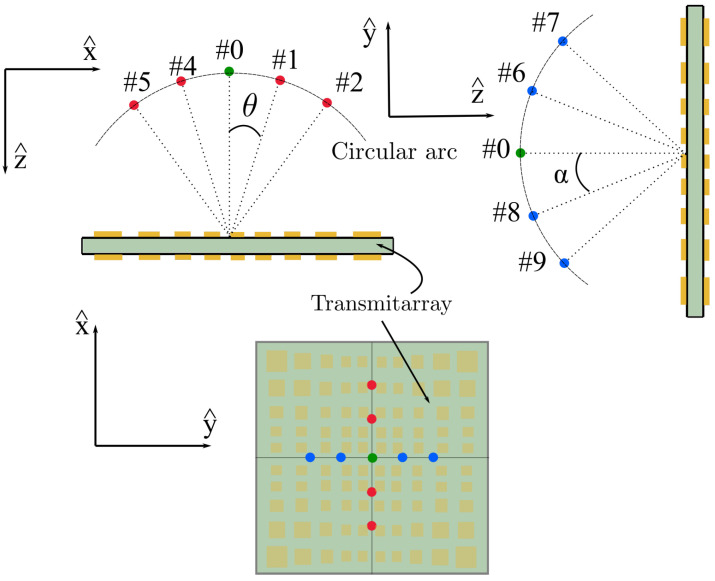
Spatial distribution of the feeds along the $\hat{x}$ and $\hat{y}$ axes.

One approach to generate a near-field multi-focusing area is based on increasing the number of feeds, so that each feed is associated with one focused spot. If the elements are considered ideal phase shifts, the amplitude of the transmitted field of a transmitarray only depends on the amplitude of the incident field, whereas the phase on two factors: (1) the phase shift of the cell and (2) the phase of the incident field [19]. Therefore, if the tapers of the different incident fields are nearly similar and the cells are designed to ensure a good angular stability, the distance and the direction of the focused spot are given by the phase response of the cell and the phase of the incident field, respectively. If a transmitarray is designed to focus using a central feed configuration, whenever the additional feeds radiate a similar incident field over the antenna surface, different spots will be created with a direction specified by each incident phase. Hence, it must be guaranteed that the distance between the phase center of each feed and the transmitarray is preserved independently of the feed position. A location that satisfies this condition independently of the number of feeds is a parabolic distribution. However, in this case, it is simplified to an arc defined by the midpoint of each transmitarray edge and the central feed, as is shown in Figure 1. The error considering this arc approach is low since the parabola is close to a circular-arc path within the proposed angles. Note that the feeds are always pointing to the geometric center of the transmitarray to ensure a similar amplitude on the incident field.

The distance between adjacent feeds is defined by the angle α and θ (see Figure 1) for the cut xoz and yoz, respectively. Setting the center of the coordinates at the geometric center of the transmitarray, the central feed is at $\left(\hat{x},\;\hat{y},\;\hat{z}\right)\;=\;\left(0,\;0,-r\right)$ mm, and the other feeds are located at:

(1) $$\begin{array}{cc} \left(\hat{x},\;\hat{y},\;\hat{z}\right) & \;=\;(-r\sin\theta ,\;0,-r\cos\theta )\;\mathrm{for}\;\mathrm{the}\;\mathrm{xoz}\;\mathrm{cut}\\ \left(\hat{x},\;\hat{y},\;\hat{z}\right) & \;=\;(0,-r\sin\alpha ,-r\cos\alpha )\;\mathrm{for}\;\mathrm{the}\;\mathrm{yoz}\;\mathrm{cut} \end{array}$$

where *r* is the radius of the arc where the feeds are located, defined by the $\hat{z}$ coordinate of the central feed, and θ and α are the angles described previously.

## 3. Near-Field Scanning Transmitarray Feed by a Cluster

### 3.1. Antenna Optics and Feed Cluster

In a first approach to evaluate the viability of this concept, a cluster of five feeds is analyzed to illuminate the transmitarray through a 1D distribution along the $\hat{x}$-axis, with an angular distribution $\theta \;=\;5^{{}^{\circ}}$ and an arc of radius 100 mm. Table 1 outlines the location of the phase center of each feed in the $\hat{x}$-axis according to (1).

**Figure 2:**
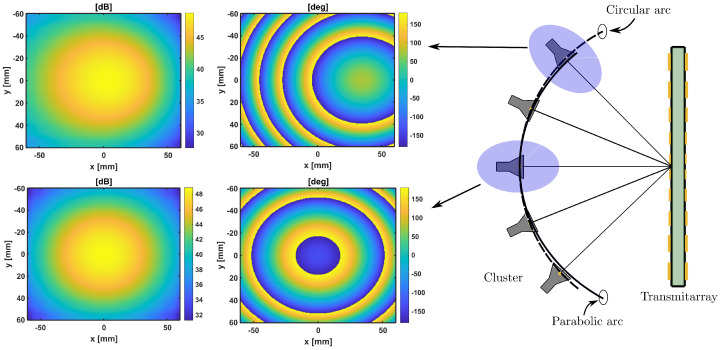
Evaluation of the incident field (amplitude (dB) and phase (deg)) for the feed at $\theta \;=\;0^{{}^{\circ}}$ and $\theta \;=\;20^{{}^{\circ}}$.

**Figure 3:**
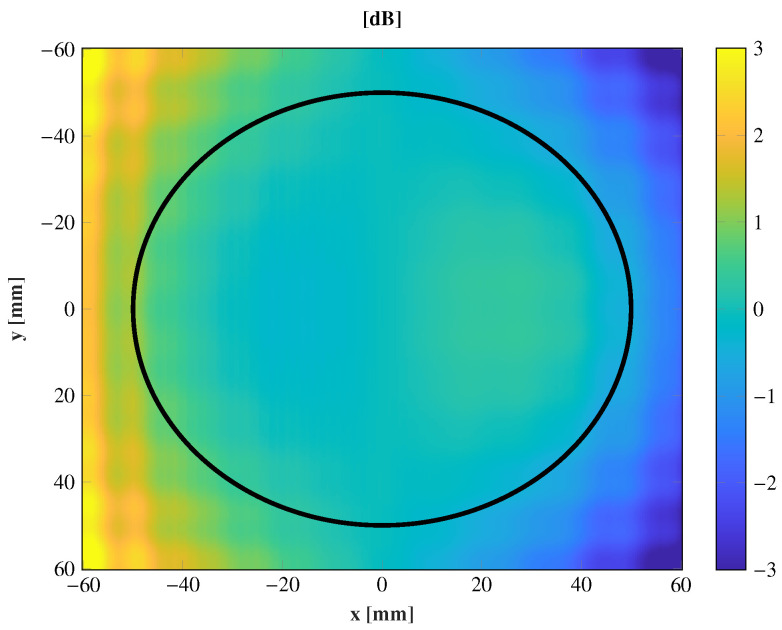
Differences of the amplitude taper (dB) on the transmitarray surface for the feeds $\theta \;=\;0^{{}^{\circ}}$ and $\theta \;=\;20^{{}^{\circ}}$. The solid black line limits the area with less than a 1 dB of difference.

This cluster feeds a transmitarray made up of 576 elements on a regular gird of 24 × 24 elements with a periodicity of 5 × 5 mm${}^{2}$ in both the $\hat{x}$ and $\hat{y}$ direction. The equivalent aperture of the transmitarray is 169.70 mm${}^{2}$ (11.2λ × 11.2λ) at the central frequency, 28 GHz. According to this configuration and using a standard gain horn of 15 dBi, the incident field is analyzed at different positions to evaluate their differences. In particular, the central position and the extreme feed are compared, which is the worst case. The comparison is carried out with a full-wave simulation using CST Microwave Studio [20]. The incident field at 28 GHz is obtained at these two positions, and the results are shown in Figure 2. Both incident fields behave similarly, and the difference is lower than 1 dB in an area of radius 50 mm (see Figure 3). These differences increase on the transmitarray edge due to the projection of the incident field on the transmitarray surface. The incident phase changes since it only depends on the phase center location. Hence, if a transmitarray is designed properly to focus the near-field on a given spot, with a single feed, it can be assumed that locating different feeds according to (Figure 1), the different incoming fields may be focused on different spots regarding the incident progressive phase variations.

### 3.2. Near-Field Focused Transmitarray

A transmitarray to focus the near-field on a single point is designed by applying geometrical optics. The phase distribution of the transmitarray elements is based on reaching a constant phase delay from the feed to the focused point. The phase delay of the transmitarray elements may compensate the different ray paths along its radius according to:

(2) $$\varphi_{inc}\left(\rho \right)\;+\;\varphi_{TA}\left(\rho \right)\;+\;\varphi_{out}\left(\rho \right)\;=\;\zeta$$

where $\varphi_{inc}\left(\rho \right)$ is the phase of the incident field at the transmitarray surface; $\varphi_{TA}\left(\rho \right)$ is the phase shift introduced by the transmitarray elements; $\varphi_{out}\left(\rho \right)$ is the phase delay because of the path from the outer transmitarray surface to the focusing point; and ζ is a constant. The radius ρ is defined as $\rho \;=\;\sqrt{x_{m}^{2}\;+\;y_{n}^{2}}$, regarding the center of the transmitarray and ($x_{m},y_{n})$ the coordinates of the center of the element. Considering ζ = 0 and applying geometrical optics, the phase shift distribution of the transmitarray elements can be computed as:

(3) $$\varphi_{TA}\left(\rho \right)\;=\;k_{0}\left(\sqrt{r^{2}\;+\;\rho^{2}}+\sqrt{F_{TA-f_{0}}^{2}\;+\;\rho^{2}}\right)$$

where $k_{0}$ is the vacuum wave number; *r* is the distance from the phase center of the feed to the center of the transmitarray; and $F_{TA-f_{0}}$ the distance from this center to the focusing point.

## 4. All Dielectric Unit Cell

### 4.1. Working Principle

Dielectric-only cells can be used to physically implement the phase shift $\varphi_{TA}\left(\rho \right)$ of a transmitarray element. These cells behave as an effective index medium adding a particular delay on the transmitted ray. The effective index medium $\eta_{eff}$ is related to the effective relative dielectric constant as $\eta_{eff}^{2}\;=\;\epsilon_{r,eff}$, and the delay introduced by the cell is computed as:

(4) $$\varphi \left(\rho \right)\;=\;\frac{2\pi f\sqrt{\epsilon_{r,eff}}}{c_{0}}t$$

where *f* is the operational frequency, $c_{0}$ is the speed of light in a vacuum, and *t* is the length of the cell.

**Figure 4:**
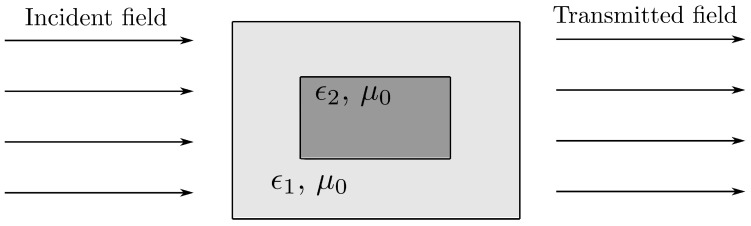
Example of dielectric cells based on the combination of two spatially uniform and isotropic materials with dielectric constants $\epsilon_{r1}$ and $\epsilon_{r2}$.

Hence, the implementation of the phase delay φ(ρ) by a cell may be done by controlling the $\epsilon_{r,eff}$ of the cell. One approach is to use dielectric cells based on the combination of two spatially uniform and isotropic materials with dielectric constant $\epsilon_{r1}$ and $\epsilon_{r2}$. If the first material hosts the second material as Figure 4 shows, the effective dielectric constant of the assembled cell only depends on $\epsilon_{r1}$ and $\epsilon_{r2}$, but not on the geometry [21,22].

The effective dielectric constant can be calculated as:

(5) $$\epsilon_{r,eff}\;=\;\left(\epsilon_{r2}\;-\;\epsilon_{r1}\right)G\;-\;\epsilon_{r1}$$

where *G* is the volume fraction of the second material over the whole volume of the assembled cell. The complexity of the cell can be reduced if one of the dielectrics is air ($\epsilon_{r2}\;=\;\epsilon_{0}$); thus, only one dielectric is needed to accomplish (5).

### 4.2. Phase Response Control with Air Inclusions

**Figure 5:**
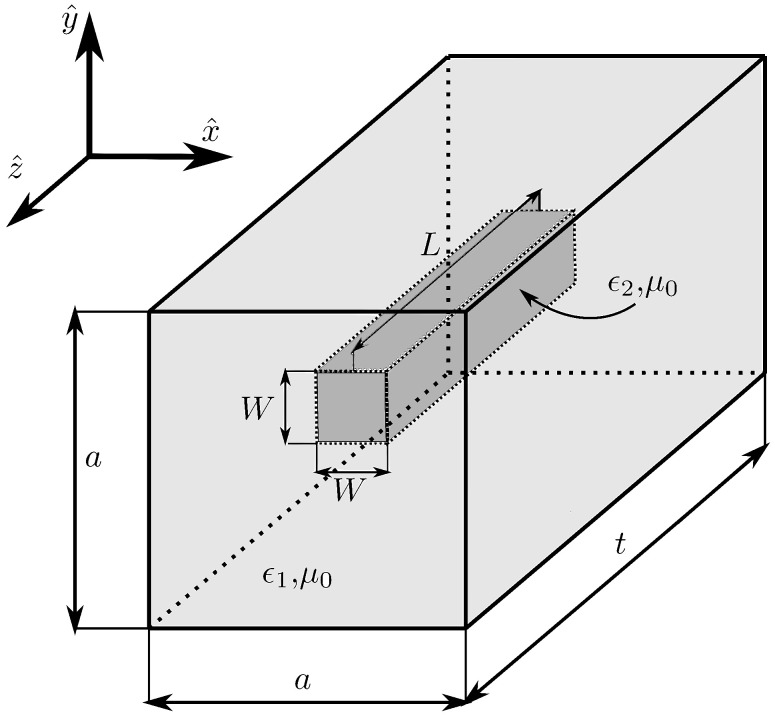
Sketch of a dielectric prism cell using air gap inclusions to change the filling of the cell and control the dielectric constant of the cell.

The proposed cell is made of poly-lactic acid (PLA) ($\epsilon_{r1}\;=\;2.85$ and tanδ = 0.0121 at 40 GHz) [23], acting as a dielectric host of an air gap inclusion [24]. The size of the inclusion controls *G*; therefore, it controls the $\epsilon_{r,eff}$ of the cell. The geometry of the dielectric cell, both the host and air gap inclusion, is a square prism of dimensions a × a × t and variable dimensions W × W × L, respectively, as Figure 5 depicts. The ratio between the air and dielectric material can be computed as:

(6) $$G\;=\;\frac{W^{2}L}{a^{2}t}$$

The cell is studied with the electromagnetic software CST Microwave Studio, setting periodic boundary conditions in the *x*- and *y*-axes and open air in the propagation direction *z*. The study is carried out for normal incidence using a plane wave with the electric field defined in the *y* direction. The air gap dimensions are swept between W ∈ (0, 5) mm and L ∈ [0, 20] mm, while the dielectric prism keeps its dimensions fixed at 5mm × 5mm × 20 mm. Applying (5) to this value, $\epsilon_{r,eff}$ varies from 1.28 to 2.85, which is a full PLA cell.

**Figure 6:**
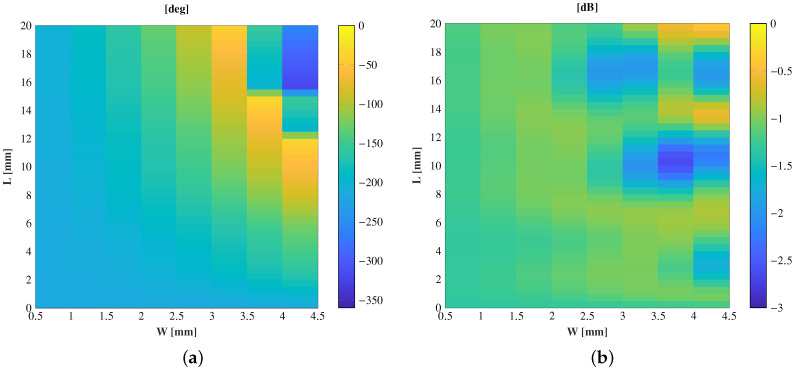
Cell response as a function of the air gap dimension at 28 GHz and normal incidence. (**a**) Phase (deg); (**b**) amplitude (dB).

The study was carried out at a central frequency of 28 GHz. The amplitude and phase response of the transmission coefficients of the cell are shown in Figure 6 as a function of the air gap dimensions for normal incidence and 28 GHz. The phase response entirely covers a $360^{{}^{\circ}}$ range, and the transmission losses are lower than 1.5 dB in more than 85% of the cells.

### 4.3. Lens Design

**Figure 7:**
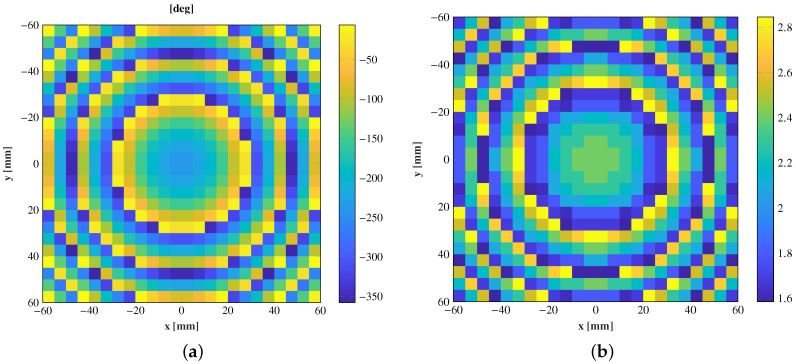
(**a**) Transmission phase shift (deg) of the transmitarray elements along the surface to focus the near-field. (**b**) Effective dielectric constant of each cell to produce the desired phase shift.

The design process of the lens is based on geometrical optics according to (2) and (3). Applying these equations to a transmitarray with the optics defined previously (r = −100 mm) (central feed) and to focus on a spot at 150 mm ($F_{TA-f_{0}}$), the phase distribution of the elements ($\varphi_{TA}\left(\rho \right)$) along the transmitarray surface of Figure 7a is obtained. The equivalent $\epsilon_{r,eff}$ of each cell is shown in Figure 7b. Then, to physically implement this phase shift, the cells have to be accurately designed. In the design process of the cells, the dimensions of the air gaps, *W* and *L*, are adjusted to introduce a phase shift φ(ρ) similar to $\varphi_{TA}\left(\rho \right)$. The adjustment is made cell-by-cell using the phase response of Figure 6b. The amplitude response is used to discard the cells with high transmission losses. The final layout is composed by the cell that minimizes the differences between its phase shift and the theoretical response of Figure 7.

**Figure 8:**
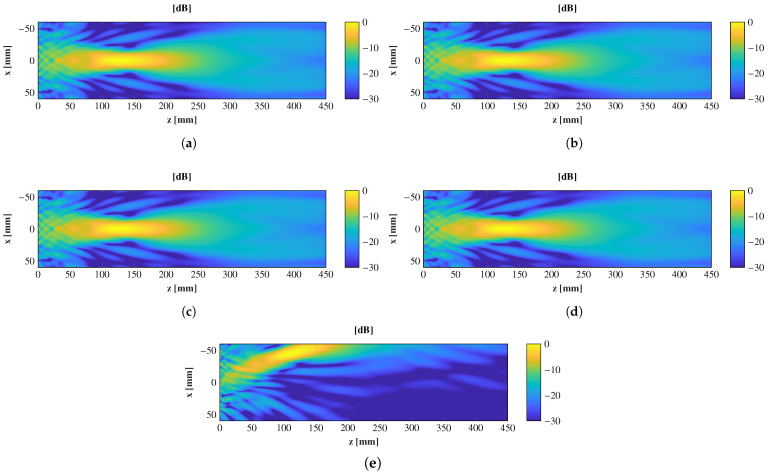
Normalized simulated amplitude electric field on the plane xoz for several positions of the feed. (**a**) $\theta \;=\;0^{{}^{\circ}}$, (**b**) $\theta \;=\;5^{{}^{\circ}}$, (**c**) $\theta \;=\;10^{{}^{\circ}}$, (**d**) $\theta \;=\;15^{{}^{\circ}}$, and (**e**) $\theta \;=\;20^{{}^{\circ}}$

**Figure 9:**
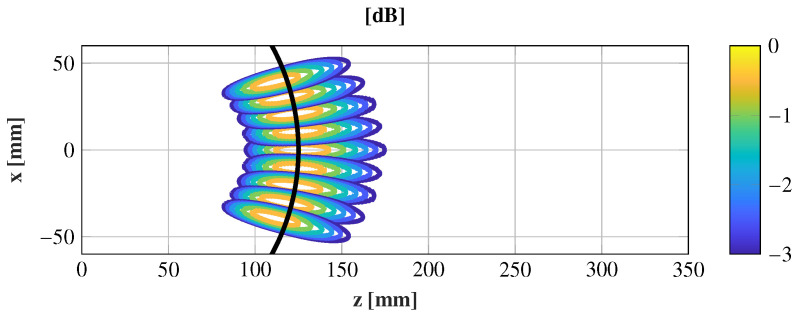
Normalized near-field focused spot comparison for several feed positions, $\theta \;\in \;{\left[0,\;20\right]}^{{}^{\circ}}$.

The entire antenna was analyzed in CST Microwave Studio in a full-wave simulation. The transmitarray is illuminated with a pyramidal horn antenna of 15 dBi gain and placed at several positions, from $\theta \;=\;0^{{}^{\circ}}$ to $\theta \;=\;\pm 20^{{}^{\circ}}$. The electric field for the xoz plane and each position is shown in Figure 8, and the near-field spots are simultaneously compared in Figure 9.

Both figures show that the transmitarray radiates a perfectly near-field spot for the central position. The transmitarray is designed to focus at z = 150 mm; even so, the maximum of the field is compressed to 125 mm. When using the conjugate phase approach, the phase of the antenna elements is in phase at the focal point, but the maximum is located at a position closer to the antenna [25]. Regarding the other feed positions, the transmitarray can generate as many spots as the feeds used, noting that there is a physical limitation due to the antenna aperture size used as the feed. Depending on the linear polarization used, this horn antenna can be placed with an angular distribution of $10^{{}^{\circ}}$ or $15^{{}^{\circ}}$. The locations of the spots are similar to the angular distribution of the feeds, and the maximum of the spots is barely compressed for angles closer than $15^{{}^{\circ}}$.

**Figure 10:**
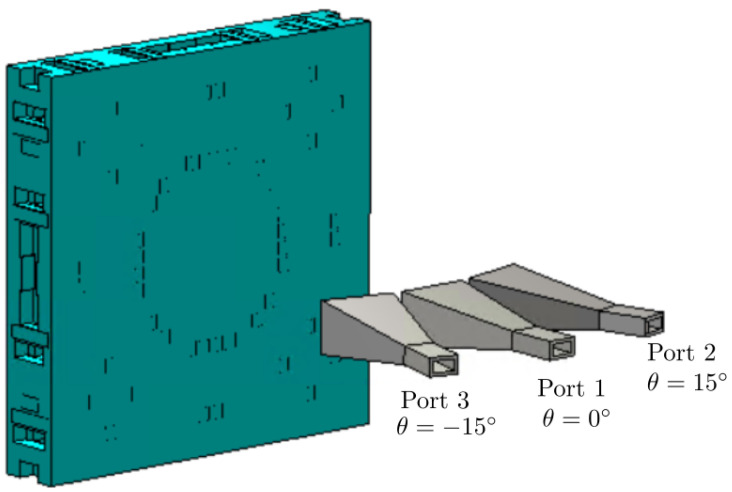
Setup to simulate the S parameters of the three horn antennas operating at the same frequency carrier.

**Figure 11:**
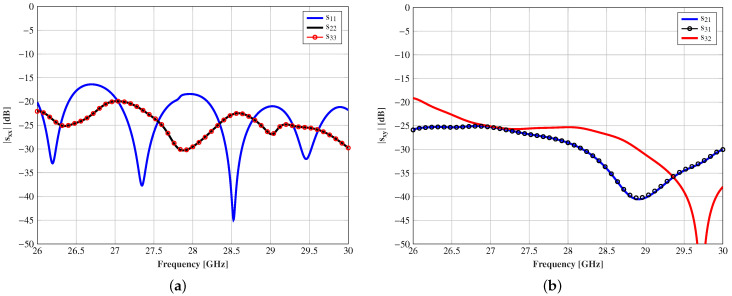
S parameters of the three horn antennas (**a**) $s_{11}$, $s_{22}$, and $s_{33}$ and (**b**) $s_{21}$, $s_{31}$, and $s_{32}$ according to the port definition given in Figure 10.

In this approach, the horn antennas are supposed to work in close, but different carriers, having a high isolation between horns. However, the isolation was also computed assuming the same frequency carrier for all the feeds, which can be considered the worst case. Figure 10 shows the setup with CST Microwave Studio to analyze the S parameters of the horn antennas, considering the lens and three horns placed at $\theta \;=\;0^{{}^{\circ}}$ (Port 1), $\theta \;=\;15^{{}^{\circ}}$ (Port 2), and $\theta \;=\;-15^{{}^{\circ}}$ (Port 3). The magnitude of $s_{11}$, $s_{22}$, and $s_{33}$ shows that the three horn antennas are matched since this parameter is below −15 dB from 26 to 30 GHz (see Figure 11a). Additionally, the magnitudes of $s_{21}$, $s_{31}$ ($s_{21}$ = $s_{31}$), and $s_{32}$ are shown in Figure 11b, and the level is almost below −20 dB in the entire band. These results show a high isolation between adjacent horn antennas working in the same frequency carrier. Note that the coupling between the edge horn antennas is slightly higher because their tilting angle is the same for both, but of the opposite sign ($\theta \;=\;\pm 15^{{}^{\circ}}$). Therefore, each edge horn antenna suffers from the specular reflection produced by the lens surface and the other edge horn antenna.

## 5. Experimental Validation

**Figure 12:**
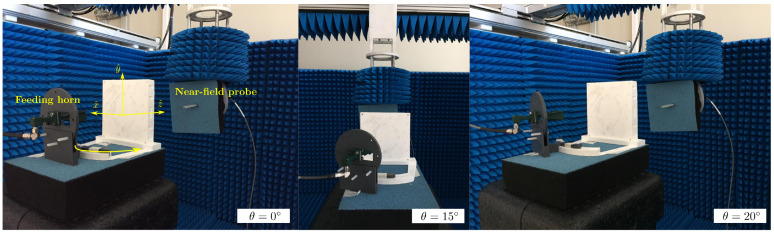
Setup used to measure the near-field radiated by the prototype in the planar acquisition range as the University of Oviedo.

The proposed planar dielectric lens made up of 576 square prism cells was manufactured and measured at the facilities at the University of Oviedo. The lens was manufactured with a 3D printing technique using the printer Ultimaker 3 and measured in a planar acquisition range. The setup in the planar range is depicted in Figure 12. A vector network analyzer PNA-X from Keysight was connected to the feeding horn, a standard gain horn of 15 dBi (Flann 22240-15), and a second port was connected to an open-ended Ka-band wave-guide used as a near-field probe. Due to the limitations of the facilities, only one horn was used in the measurements. However, the structure bearing the horn antenna enables moving the feed into an arc of radius 100 mm, thus placing the horn antenna on the different positions of the cluster. The measurements were performed at three different feed positions ($\theta \;=\;0^{{}^{\circ}}\;,15^{{}^{\circ}},\;20^{{}^{\circ}}$) in a bandwidth of 4 GHz, from 26 to 30 GHz, evaluating the electric field on the horizontal plane xoz.

**Figure 13:**
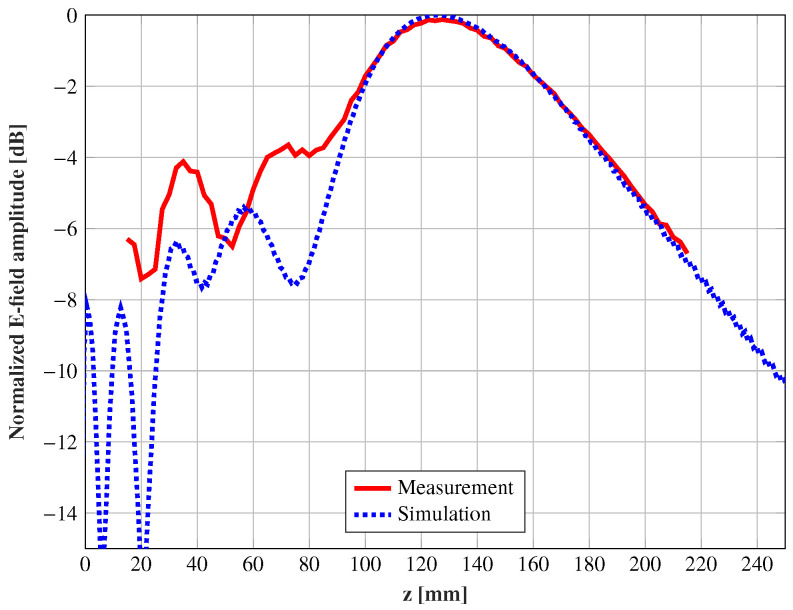
Comparison between the simulations and measurements of the normalized amplitude (dB) of the electric field along the propagation direction and x = y = 0 at 28 GHz with the feed $\theta \;=\;0^{{}^{\circ}}$.

**Figure 14:**
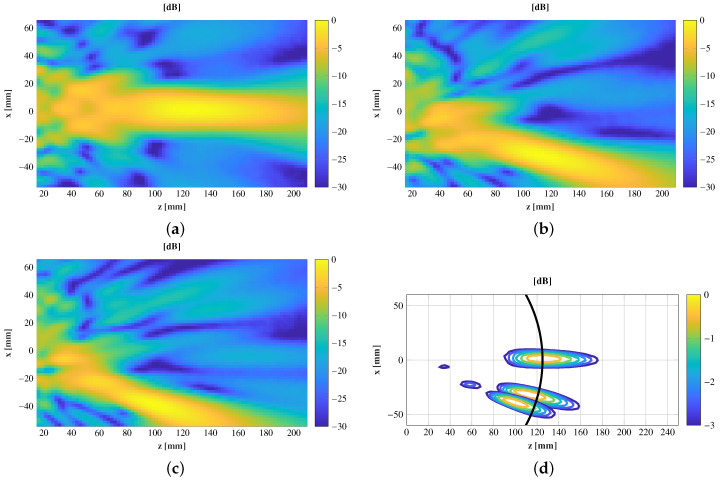
Normalized measured amplitude in dB for three different feed positions. (**a**) $\theta \;=\;0^{{}^{\circ}}$, (**b**) $\theta \;=\;15^{{}^{\circ}}$, (**c**) $\theta \;=\;20^{{}^{\circ}}$, and (**d**). Comparison among the three focused spots.

The copolar component $\hat{y}$ of the electric field through the propagation direction $\hat{z}$ and x = y = 0 is measured and compared with the simulations in Figure 13. The simulations and measurements highly agree at the maximum position and the field decay within the measured area, obtaining a similar depth-of-focus considering a 3 dB decay of the electric field. In Figure 14, the electric field in the plane xoz at three different positions ($\theta \;=\;0^{{}^{\circ}},\;15^{{}^{\circ}}\;\mathrm{and}\;20^{{}^{\circ}}$) is shown, and Table 2 outlines a comparison between the simulated and measured focusing performances. Due to the antenna symmetry, only the three positions shown in Figure 12 were measured. These results confirm that a displacement of the feed according to (1) moves the near-field spot, while the focusing characteristics remain practically unchanged. For the central spot, both the maximum location and depth-of-focus, highly agree with simulations. The agreement for the middle spot ($\theta \;=\;15^{{}^{\circ}}$) is also significantly good, highlighting that the depth-of-focus is not only close to the simulations, but also to the central spot. At the widest position ($\theta \;=\;20^{{}^{\circ}}$), an angular shift of the spot starts to appear, and the position of the maximum is compressed. These effects are a consequence of the phase aberrations due to the off-focus feeding.

**Figure 15:**
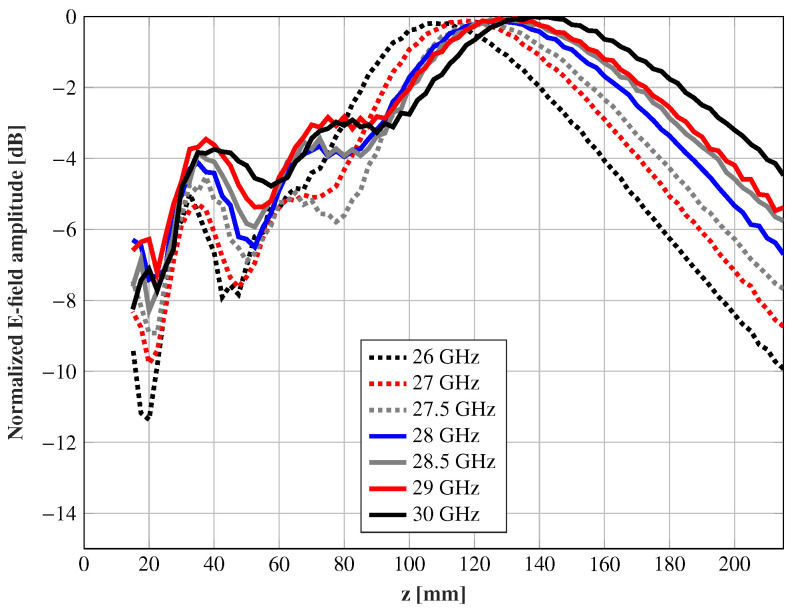
Measurements normalized to their maximum values of the amplitude (dB) of the electric field intensity along the propagation direction and x = y = 0 at different frequencies for the feed $\theta \;=\;0^{{}^{\circ}}$.

The in-band response is analyzed from 26 to 30 GHz. In Figure 15, the normalized electric field amplitude through the propagation direction and x = y = 0 is shown for the central spot and different frequencies. The measurements presented an extremely stable behavior from 27.5 to 29 GHz, keeping a similar depth-of-focus, and the maximum barely shifted it position. At the frequencies out of this range, the maximum of the field was displaced, whilst the depth-of-focus was enlarged or shortened, regarding the frequency. These results show a great in-band response compared with transmitarrays based on resonant elements.

In Table 3, different published works of antennas with scanning or multi-focusing capabilities are compared with this work. These solutions are typically used in far-field (FF) applications, while only a few published works may be found on near-field (NF) ones. In [18], a dielectric transmitarray was proposed to reach a multi-focusing solution, in particular to simultaneously radiate four focused spots. The transmitarray was fed by a single feed; thus, the spots should be far from each other with a broad angular deviation to avoid interferences among them. Therefore, this solution is not suitable for closed spots. In addition, the transmitarray design was based on a subdivision of the antenna aperture in order to focus one spot per subdivision. This technique normally requires oversized apertures and obtains fixed spots, but not scanning performances. However, the proposed technique can be used to obtain either fixed spots or a scanning. Hence, this work proposes an alternative for multi-focusing within the near-field with scanning capabilities since the spot positions regard the feed placement. Additionally, the spots can be generated as close as the physical dimensions of the feeds enable with a high isolation between feeds. This approach can be applied at least in a scanning range of $40^{{}^{\circ}}$, keeping the focusing properties on all the spots in a broad-band solution in the Ka-band due to all the dielectric cells.

## 6. Conclusions

A total dielectric transmitarray was analyzed to generate a multi-focusing area in the near-field of the antenna in the Ka-band. The design process of the transit array is based on geometrical optics and considers a single feed, in central configuration, to focus the near-field on a given spot. Then, the number of feeds is increased to obtain a transmitarray fed by a cluster of horns placed along a circular distribution in the horizontal main plane of the transmitarray. The location of the feed is carefully chosen to keep the same distance from each phase center to the center of the transmitarray. This configuration guarantees obtaining a very similar amplitude in the incident field whilst the phase changes and determines the direction of the spot radiated. In order to verify this concept, a transmitarray is designed using dielectric-only cells. These cells are based on a dielectric and air inclusions that control the filling of the cell and, therefore, change the phase shift of the cell. The design is full-wave simulated, giving successful results as the transmitarray not only focuses the central spot, but also the other incoming fields provided by the cluster. In light of these results, the transmitarray was manufactured using a 3D printing technique, and an easily manufactured and low-cost prototype was obtained. The prototype was measured in a planar acquisition range at three different feed locations. The measurements showed a high agreement with simulations in terms of the focused spots performances (DoF and maximum location). Additionally, it was verified that the proposed feed distribution generates one spot by each feed, which should use different shifted carriers or time slots in order to avoid interferences. The prototype radiates similar near-field focused spots within an angle of $\pm 20^{{}^{\circ}}$, where only at the widest positions, the spot starts to be defocused. Additionally, the in-band response of the transmitarray shows a very stable behavior in the whole Ka-band, especially from 27.5 to 29 GHz. In light of these results, only-dielectric transmitarrays fed by a cluster can be a potential alternative for the generation of multi-focusing near-field spots at the millimeter band.

## Figures and Tables

**Table 1:** Phase center location for different θ according to (1).

Feed	$\theta \;=\;\pm 0^{{}^{\circ}}$	$\theta \;=\;\pm 5^{{}^{\circ}}$	$\theta \;=\;\pm 10^{{}^{\circ}}$	$\theta \;=\;\pm 15^{{}^{\circ}}$	$\theta \;=\;\pm 20^{{}^{\circ}}$
$\hat{x}$	0	±8.71	±17.36	±25.88	±34.22
$\hat{y}$	0	0	0	0	0
$\hat{z}$	−100	−99.61	−98.48	−96.59	−93.97

**Table 2:** Comparison of the maximum location (z, x) and the depth-of-focus (DoF) between the simulations and measurements.

Feed (θ (deg))	Simulations		Measurements	
	Maximum (mm)	DoF (mm)	Maximum (mm)	DoF (mm)
$\theta \;=\;0^{{}^{\circ}}$	(125.64,0)	79.95	(130,0)	85
$\theta \;=\;15^{{}^{\circ}}$	(116.60,−30)	76.11	(112.5,−32.5)	75.76
$\theta \;=\;20^{{}^{\circ}}$	(110.60,−38.89)	74.20	(97.5,−49.5)	65.62

**Table 3:** Comparison of the proposed antenna with other published works. TA, transmitarray; FZP, Fresnel zone plate.

Work	Type of Antenna	Scanning (S) or Multi-Focusing (MF)	Far-Field (FF) or Near-Field (NF)	Technique	Unit Cell	Range	Frequency
[13]	Lens	S	FF	Multiport feeder	Dielectric	50${}^{{}^{\circ}}$	24–28 GHz
[16]	TA	S	FF	In-plane feeder displacement	Metal dielectric stacks	100${}^{{}^{\circ}}$	20 and 30 GHz
[17]	TA	S	FF	Transmitarray rotation	Dielectric	54${}^{{}^{\circ}}$	30 GHz
[12]	FZP	S	NF	In-plane feeder displacement	Metallic rings	N.A.	32 GHz
[18]	TA	MF	NF	Aperture division with different focus points	Dielectric	Only 4 spots	35 GHz
This work	TA	MF/S	NF	Cluster of feeders	Dielectric	40${}^{{}^{\circ}}$	26–30 GHz

## Data Availability

Data sharing not applicable No new data were created or analyzed in this study. Data sharing is not applicable to this article.
